# Short-Chain Fatty Acid Utilization in *Cyberlindnera jadinii* for Single-Cell Protein and Odd-Chain Fatty Acid Production

**DOI:** 10.3390/microorganisms13071558

**Published:** 2025-07-02

**Authors:** Christian Hermansen, Rowanne Siao, Gi Gi Chua, Mikko Ru Xuan Lee, Aaron Thong, Melanie Weingarten, Nic Lindley, Eric Charles Peterson

**Affiliations:** 1 Singapore Institute of Food and Biotechnology Innovation (SIFBI), Agency for Science, Technology, and Research (A*STAR), 31 Biopolis Way, Nanos, Singapore 138669, Singapore; 2 Institut National de la Recherche Scientifique—Eau Terre Environnement (INRS-ETE), 490 Rue de la Couronne, Quebec City, QC G1K 9A9, Canada

**Keywords:** short-chain fatty acid, non-conventional yeast, *Cyberlindnera jadinii*, bioprocess development, single-cell protein, single-cell oil, odd-chain fatty acids, food, feed

## Abstract

In view of the growing global need for sustainable protein sources, this study explores the utilization of short-chain fatty acids into single-cell protein using the non-conventional yeast *Cyberlindnera jadinii*. Short-chain fatty acids can be sustainably produced via anaerobic digestion of organic waste, presenting a promising fermentation substrate for a circular bioeconomy. *Cyberlindnera jadinii* is demonstrated to be capable of growing on acetate, propionate and butyrate as both a carbon and energy source without strong inhibition. Bioprocess development was conducted in stirred tank bioreactors, where a fed-batch pH-stat bioprocess led to improved efficiency without substrate inhibition. The highest titer of 31.3 ± 1.0 g/L, rate of 0.67 ± 0.02 g/L/h and yield of 0.36 ± 0.01 g/g was achieved with propionate. The resulting biomass contained 41.3% crude protein, and 17.3% crude lipids with 81% unsaturated fatty acids. In contrast to acetate and butyrate, propionate as a substrate led to accumulation of 37% odd-chain fatty acids with titer, rate and yield of 1.74 ± 0.06 g/L, 0.037 ± 0.001 g/L/h and 0.020 ± 0.001 g/g. These findings confirm that short-chain fatty acids are viable fermentation substrates not only for single-cell protein, but also unsaturated and odd-chain fatty acid production with *Cyberlindnera jadinii*.

## 1. Introduction

The escalating global demand for sustainable protein sources has driven the exploration of alternative biotechnological solutions, particularly those that align with circular bioeconomy principles [1,2]. Single-cell protein (SCP) derived from microbial biomass has emerged as a promising avenue for addressing both food security and environmental sustainability, owing to its high protein content, rapid microbial growth rates, and ability to utilize diverse, low-cost substrates [3,4,5,6]. Among the potential feedstocks, short-chain fatty acids (SCFAs), most notably acetate, propionate, and butyrate, offer significant promise due to their renewable origin from the anaerobic digestion of organic waste streams [7,8]. Typical concentration ranges of SCFAs in anaerobic digestion are 100–2000 mg/L for acetic acid, 10–1000 mg/L for propionic acid, and 10–500 mg/L for butyric acid [9]. Various techniques can be employed to promote higher concentrations of total and specific SCFAs [10].

In contrast to traditional fermentation substrates such as sugars and glycerol, acetate does not go through glycolysis, but enter central metabolism as acetyl-CoA, the key precursor for, e.g., biomass and lipid production, potentially offering a high yield per carbon-atom basis [5]. For these reasons acetate has been explored for recombinant protein [11], lipid [12,13], consumer care [14], pharmaceutical [13] and biopolymer products [15,16]. The unique metabolic pathways of other SCFAs lend themselves to production of specific classes of compounds. Butyrate has mainly been explored for poly(hydroxybutyrate) production, for which it is a direct precursor [17]. Propionate contains three carbon atoms, and acts as a precursor for biosynthesis of odd-chain fatty acid (OCFA) [18,19]. Recently the use of mixed SCFAs has received attention for microbial oil production in yeast [8], including OCFA from anaerobic digestate containing propionate [20]. These uses underscore the potential of SCFAs as sustainable feedstocks in a circular bioeconomy [21]. However, establishing efficient bioprocesses remains challenging due to their toxicity [22].

The yeast *Cyberlindnera jadinii* (formerly referred to as *Candida utilis*, the anamorph state) is a non-conventional yeast and generally regarded as safe (GRAS) organism with established applications in animal feed and SCP production [23,24,25,26]. Most studies have focused on the utilization of various carbohydrate-based substrates for SCP production, such as glucose, xylose, cellobiose, mannose and sucrose [27,28], some of which can be derived from lignocellulosic biomass including spruce wood [29,30] and oil-palm empty fruit bunch [31]. However, the ability of *C. jadinii* to metabolize SCFAs for biomass and lipid production remains underexplored [32].

Beyond protein, *C. jadinii* may yield unsaturated fatty acids [33,34] and OCFAs [35,36]. Unsaturated fatty acids, including oleic acid (C18:1), linoleic acid (C18:2) and linolenic acid (C18:3), together with OCFAs such as pentadecanoic (C15:0) and margaric (C17:0) acids, offer nutritional and functional advantages in food and feed applications. Unsaturated fatty acids improve lipid profiles, lowering the risk of cardiovascular disease and type 2 diabetes mellitus [37], and enhance meat and egg quality by enriching products in health-promoting ω-3 fatty acids [33]. Similarly, OCFAs have also been linked to reduced risk of both type 2 diabetes and cardiovascular disease, by acting as an anti-inflammatory agent and other mechanisms, and associated with lower mortality [35,38]. OCFAs also improve growth performance and intestinal morphology in livestock [39]. Integrating these bioactive lipids into SCP product can therefore boost both the nutritional value and functional performance of microbial biomass for human and animal nutrition.

In this study we investigate the capability of *C. jadinii* to grow on the SCFAs acetate, propionate, and butyrate as sole carbon sources and energy sources. We investigate SCFA toxicity to *C. jadinii* through accurate measurements of maximum specific growth rate (SGR), then develop an innovative fed-batch pH-stat bioprocess for efficient utilization of SCFAs, assessing their performance in terms of, titer, rate, yield (TRY), and biomass composition. The influence of the type of SCFA on crude protein and crude lipid contents, as well as the fatty acid profile, with special focus on the formation of OCFAs under propionate-based growth. These findings provide new insights into the use of SCFAs for SCP and lipid production in *C. jadinii*, expanding the use-cases of this non-conventional yeast, and contribute to the development of sustainable bioprocesses for food and feed applications.

## 2. Materials and Methods

### 2.1. Seed Cultures and Media

*Cyberlindnera jadinii* (ATCC 9950) was cultivated on yeast extract–peptone (YP) media including the following ingredients: yeast extract (10 g/L), peptone (20 g/L), sodium acetate, propionate or butyrate (20 g/L) and adjusted to pH 7.0 with sodium hydroxide (2 M) then autoclaved at 121 °C for 15 min. Seed cultures of 50 mL volume were incubated overnight for 24 h at 28 °C and 250 rpm shaking in 250 mL shake flasks. Seed cultures were inoculated with a single colony obtained from a yeast extract–peptone–dextrose (YPD) agar plate streaked with glycerol stock of the pure culture. Pure cultures were prepared from YPD broth cultivations with 25 g/L glucose, incubated under the same conditions as the seed cultures, and maintained at −80 °C with 50% (*v*/*v*) glycerol. YPD agar and broth were only used for preparing seed and pure cultures, not for fermentations with short-chain fatty acids, for which glucose was excluded (i.e., YP). All chemicals and ingredients were purchased from Sigma-Aldrich (Burlington, MA, USA).

### 2.2. Small-Scale Cultivation

Small-scale cultivations on increasing concentrations of acetate, propionate and butyrate were conducted in a personal bioreactor system RTS-1 (BioSan, Riga, Latvia). The 20 mL YP medium with sodium acetate, propionate or butyrate (2.5 g/L, 5.0 g/L and 10 g/L) was sterile filtered (Thermo Fisher Scientific, Waltham, MA, USA) and filled in pre-sterilized 50 mL tubes (TubeSpin^®^ Bioreactor 50, BioSan, Riga, Latvia). Cultivation was performed in biological triplicates at 28 °C and 1500 rpm over a period of 72 h, with online monitoring of optical density at 850 nm (OD850). The SGR was derived by logarithmic transformation of the OD850 trend, and linear fitting by least squares methodology in Excel Version 2504 (Microsoft, Redmond, WA, USA) to the linear segment 3 and 6 h cultivation time, excluding early data points due to potential lag-phase and instrument measurement precision.

### 2.3. Stirred Tank Bioreactor Fermentation

Volumes of 200 mL YP media containing either sodium acetate, propionate or butyrate as the carbon source were prepared in 500 mL bioreactors (Getinge Applikon, Delft, The Netherlands). The bioreactors were autoclaved as described above. Bioreactors were operated at 28 °C, with air sparging of 200 mL/min and agitation of 1000 rpm to 2000 rpm controlled by a dissolved oxygen (DO) cascade with a set-point of 10%. Following equilibration of temperature and DO, the bioreactors were inoculated with seed cultures normalized to 0.1 optical density at 600 nm (OD600). The pH was controlled via automatic acid addition via an integrated proportional–integral–derivative (PID) controller with 0.1 pH unit deadband. Feeding of either 1 M HCl, 3 M SCFA or 5 M SCFA depending on the experiment would occur in pulses when measured pH was 0.1 pH units higher than the set-point. Pumps were volumetrically calibrated prior to the experiment and a total reservoir volume of 100 mL acid was provided.

Seed culture was inoculated to a normalized OD600 of 0.1 once bioreactor conditions were stable, with a typical seed culture OD600 of 4.0 and inoculation volume of 5 mL, and 1 mL of Antifoam 204 (Sigma-Aldrich, Burlington, MA, USA) was dosed at the start of the fermentation to prevent foaming. Samples were measured for OD600 and SCFA concentration via HPLC. After 48 h, reactor contents were harvested, then samples taken for cell dry weight (CDW) determination. In addition, 50 mL aliquots were centrifuged for 15 min at 3900 rpm, decanted, washed with normal saline, resuspended and centrifuged again. The resulting biomass pellets were frozen at −80 °C overnight then freeze-dried for 48 h (Freezone 2.5; Labconco, Kansas City, MO, USA) and used for crude protein and lipid analysis.

### 2.4. Optical Density and Cell Dry Weight

Optical density was measured at 600 nm (OD600) in triplicate by diluting 10 to 50 times with the YP media, and values accepted within the linear range of 0.2 to 0.8 absorbance units. Cell dry weight (CDW) was conducted in triplicate from 5 mL aliquots dosed in pre-weighed centrifuge tubes, centrifuged for 5 min at 13,000 rpm. The supernatant was decanted and the pellet dried in the oven at 60 °C for 48 h followed by gravimetric analysis.

### 2.5. Short-Chain Fatty Acid Quantification

Acetic acid, propionic acid and butyric acid were quantified by high-performance liquid chromatography (Agilent Infinity II 1260 HPLC; Agilent Technologies, Santa Clara, CA, USA) using a diode array detector (G7115A DAD; Agilent Technologies, Santa Clara, CA, USA) and refractive index detector (G7162A RID; Agilent Technologies, Santa Clara, CA, USA). In short, 2 mL liquid sample was filtered through 0.45 µm hydrophilic filters (Sartorius, Göttingen, Germany), then 20 μL was injected into the HPLC for analysis. SCFAs were separated on an ion-exchange column (Aminex HPX-87H; 300 × 7.8 mm, 35 °C; Bio-Rad, Hercules, CA, USA), utilizing a mobile phase of 10 mM H_2_SO_4_ at 0.6 mL/min, and detected with RID (30 °C, positive polarity). SCFAs were quantified by comparison to standard calibration curves via the OpenLab CDS Chemstation edition (C.01.07, Agilent Technologies, Santa Clara, CA, USA). Fermentable sugars and other organic acids including citric acid and lactic acid are readily analyzed using this method but were not detected unless otherwise reported.

### 2.6. Crude Protein Analysis

Crude protein was determined on 100 mg freeze-dried biomass samples wrapped in tin foil via the Dumas method (DIN EU ISO 16634) [40] with a DumaTherm (Gerhardt, Königswinter, Germany) validated by using EDTA as a QC check during each run to ensure instrument performance. Oxygen dosing was set at 400 mL/min and the combustion temperature at 990 °C. The method determines the total nitrogen content of the sample, and crude protein was then calculated using a protein conversion factor of 6.25.

### 2.7. Lipid Analysis

For lipid analysis, 1 mL hydrochloric acid solution (8.3 M) was added to 200 mg freeze-dried biomass in a screw-capped glass vial, and 10 mg of pyrogallic acid was added to minimize oxidation during analysis. The solution was heated to 70 °C with intermittent shaking for 40 min. The hydrolysates were subsequently vigorously agitated with 10 mL diethyl ether and petroleum ether (1:1) followed by separation and evaporation of the solvent prior to gravimetric analysis of total lipid content. For compositional lipid analysis, dried fats were methylated to fatty acid methyl esters (FAMEs) by combining samples with 2 mL BF_3_ (14% in methanol) and heating at 100 °C for 45 min, followed by extraction with 1 mL hexane for injection into the GC. FAMEs were analyzed by gas chromatography equipped with a flame ionization detector (FID) and a fused silica capillary column (Supelco SP2560; 100 m × 0.25 mm × 0.20 μm; Supelco, Bellefonte, PA, USA), with helium for a carrier gas (1.0 mL/min.). A sample volume of 1 μL was injected and split at 25:1, with injector and detector temperatures set at 220 °C and 250 °C, respectively. Data were processed using OpenLAB CDS Chemstation software (C.01.10; Agilent Technologies, USA).

## 3. Results

### 3.1. Small-Scale Cultivation with Increasing SCFA Concentrations

**Figure 1:**
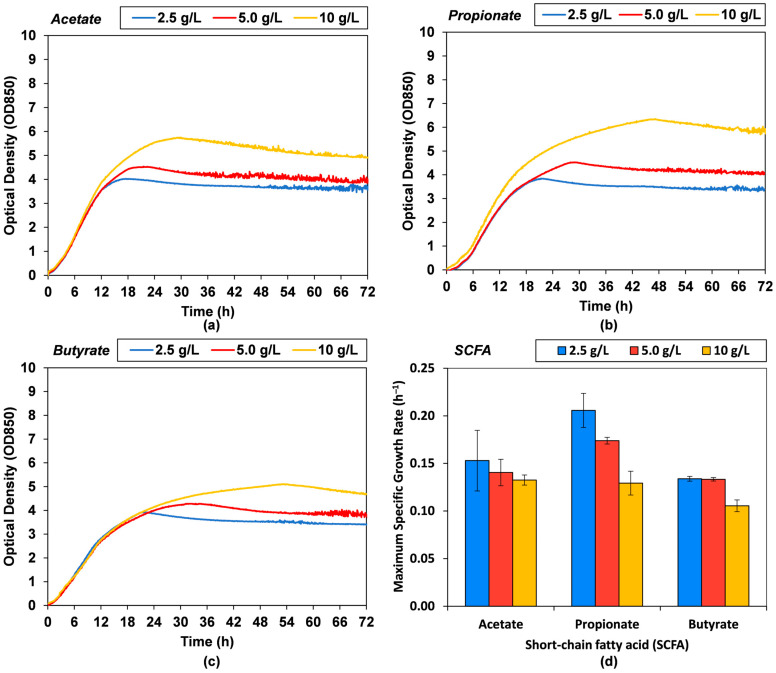
Small-scale cultivation of *C. jadinii* at increasing concentrations of short-chain fatty acids (SCFAs): (**a**) optical density measured at 850 nm (OD850) for cultivation with acetate; (**b**) OD850 for cultivation with propionate; (**c**) OD850 for cultivation with butyrate; and (**d**) maximum specific growth rate (SGR) determined from the exponential growth phase between 3 h and 6 h cultivation time.

The feasibility and potential toxicity of acetate, propionate and butyrate short-chain fatty acids (SCFAs for single-cell protein (SCP production with *Cyberlindnera jadinii* was investigated in small-scale cultivations based on online measurement of optical density (OD850). The growth can be observed to slow down around the 6 to 12 h mark, depending on the SCFA type and concentration, and typically stalls after 24 h as seen in Figure 1.

The maximum specific growth rate (SGR) was derived from the early time-point data (between 3 h and 6 h) where substrate is in excess, and growth was observed to be exponential. Least squares fit of log-linear transformation had coefficients of determination (*R*^2^) ranging from 0.96 to 1.00. Acetate and butyrate give similar maximum SGR at all concentrations, without significant decrease on increasing SCFA concentration, with maximum SGR of 0.13 ± 0.01 h^−1^ and 0.11 ± 0.01 h^−1^ for 10 g/L acetate and butyrate, respectively. In contrast, propionate led to a higher maximum SGR of 0.21 ± 0.02 h^−1^ at 2.5 g/L, decreasing proportionally to 0.17 ± 0.00 h^−1^ at 5.0 g/L and 0.13 ± 0.01 h^−1^ at 10 g/L. This decrease in SGR suggests observable substrate inhibition, which could be either from the substrate, or the increased osmotic stress from elevated ion concentrations through proportional pH control as substrate increases.

The maximum OD850 increased with SCFA concentration, and at 10 g/L was 5.8 ± 0.2 for acetate, 6.3 ± 0.1 for propionate, and 5.1 ± 0.10 for butyrate. These results indicate that SCFAs are promising substrates for *C. jadinii* cultivation. Propionate especially stands out as leading to the highest maximum SGR and maximum OD850.

### 3.2. Stirred Tank Bioreactor Fermentation with Different pH Control Settings

**Figure 2:**
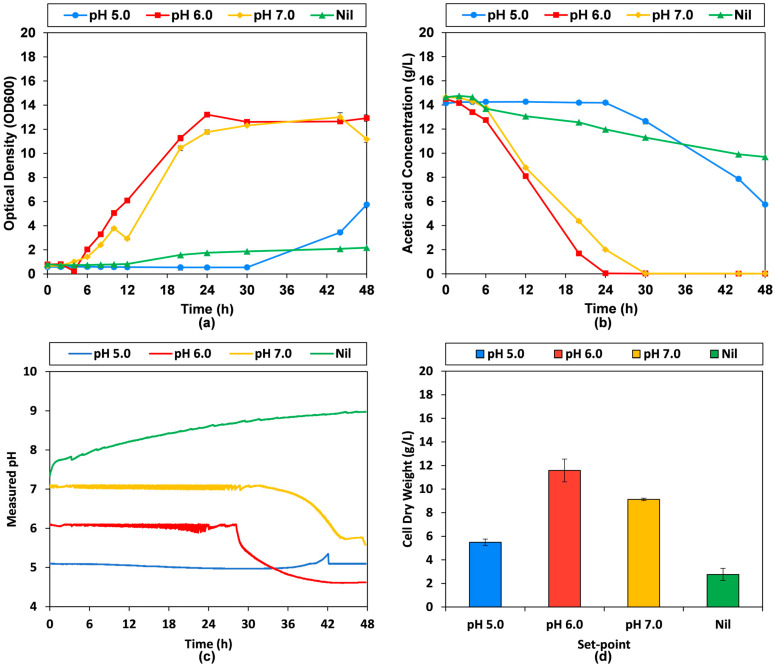
Batch bioreactor fermentation with *C. jadinii* cultivated on acetate with different pH control settings: (**a**) optical density at 600 nm (OD600); (**b**) acetic acid concentration in acid form; (**c**) measured pH during fermentation; (**d**) final cell dry weight concentration measured gravimetrically.

The pH dependence of the bioprocess was investigated in stirred tank bioreactors with pH setpoint at 5.0, 6.0, 7.0 or nil (i.e., no set-point) with 20 g/L sodium acetate (equivalent to 14.6 g/L acetic acid). The results in Figure 2 show that the set-point at pH 5.0 led to a stable pH, but relatively poor growth and substrate uptake, especially in the first 24 h of fermentation, and the cell dry weight of 5.5 ± 0.3 g/L after 48 h. The set-point at pH 6.0 performed the best, with rapid growth and substrate uptake, depleting the acetic acid by 24 h. Following substrate depletion, pH trended downwards to 4.6 at the end of 48 h, while producing 11.6 ± 1.0 g/L cell dry weight. The decreasing pH on substrate depletion is likely caused by a loss of buffering capacity in the system and accumulation of HCl from pH adjustment. No acidic by-products from metabolism were observed via HPLC. The set-point of pH 7.0 performed similar to pH 6.0, but slightly worse overall. The growth trend by OD600 was similar, while substrate uptake was slightly lower, depleting acetic acid by 30 h. The pH similarly dropped after substrate depletion, with a final value of 5.6. The final cell dry weight after 48 h was lower at 9.1 ± 0.1 g/L biomass. At a pH setpoint of 5.0, *C. jadinii* showed delayed growth, with a pronounced lag phase and decreased growth (5.5 g/L ± 0.3 g/L), clearly demonstrating the lower pH tolerance in this system. Similarly, Nil, or no set-point, led to poor outcomes, with poor growth by OD600, low substrate uptake and the lowest cell dry weight of 2.8 ± 0.5 g/L after 48 h fermentation. This poor growth is matched by steady increase in pH from the initial 7.35 of the YP medium, increasing to 9.0 at the end of 48 h due to the consumption of acetic acid by *C. jadinii*. In combination with the relatively improved performance of *C. jadinii* at pH 6, these results suggest the preferential uptake or passive diffusion across the cell membrane of the undissociated form (i.e., acetic acid) over the conjugate base, with pH rising as the equilibrium shifts to replace undissociated acid levels. As a result, this suggests the interesting possibility of using SCFAs in their acid form for both pH-control and fermentation substrates in a pH-stat.

### 3.3. The Stirred Tank Bioreactor pH-Stat Bioprocess

**Figure 3:**
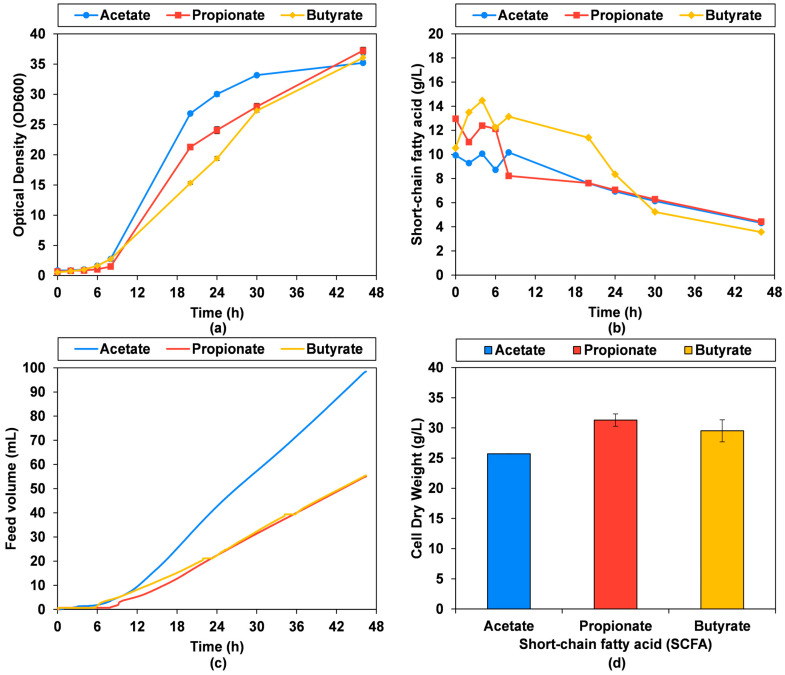
Fed-batch pH-stat bioreactor fermentation with *C. jadinii* with different SCFA substrates with 20 g/L sodium salt SCFA starting medium concentration and 5 M SCFA feed controlling pH to at the set-point of 6.0: (**a**) optical density at 600 nm (OD600); (**b**) SCFA concentration in acid form; (**c**) volume of 5 M SCFA added; (**d**) final cell dry weight concentration measured gravimetrically.

**Figure A1:**
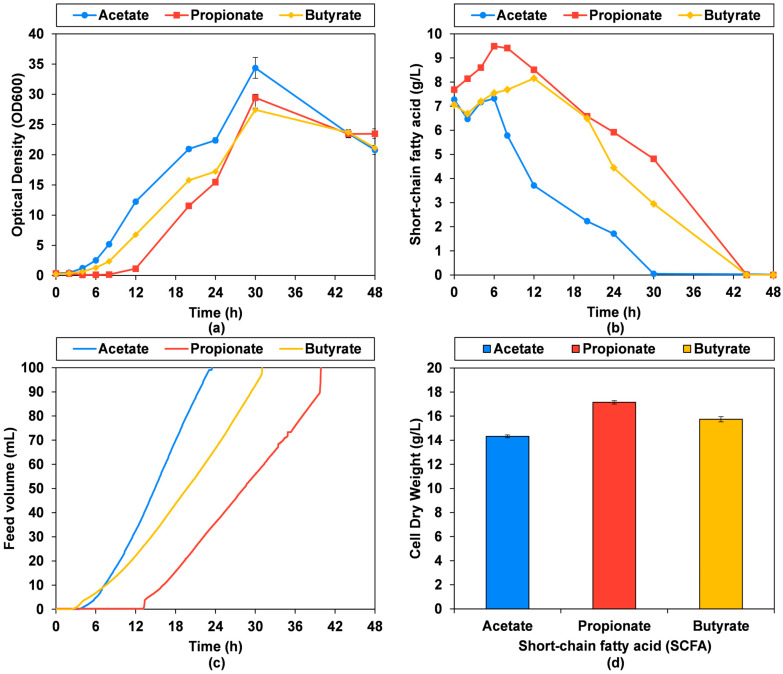
Fed-batch pH-stat bioreactor fermentation with *C. jadinii* with different SCFA substrates with 10 g/L sodium salt SCFA starting medium concentration and 3 M SCFA feed controlling pH to at the set-point of 6.0: (**a**) optical density at 600 nm (OD600); (**b**) SCFA concentration in acid form; (**c**) volume of 3 M SCFA added; (**d**) final cell dry weight concentration measured gravimetrically.

A pH-stat bioprocess was first evaluated with starting concentrations of 10 g/L sodium acetate, propionate and butyrate, and 3 M acetic acid, propionic acid and butyric acid feed to maintain a pH set-point of 6.0 ± 0.1. These conditions fully depleted the available 100 mL SCFA feed reservoir within 40 h, with concurrent cessation of growth as reported in Figure A1. These results suggest that higher concentrations of SCFA could favorably be used in the bioprocess, and therefore starting conditions of 20 g/L sodium acetate, propionate and butyrate, and feeds of 5 M acetic acid, propionic acid and butyric acid were subsequently trialed (Figure 3).

As depicted in Figure 3, the OD600 demonstrates a range of growth distributions across the three tested SCFAs, showing a faster increase for acetate, followed by propionate, and butyrate, respectively. However, final OD600 values were slightly higher for propionate and butyrate, with end-point OD600 values of 37.3 ± 0.6 and 36.1 ± 0.5, respectively, compared to 35.2 ± 0.1 for acetate. The SCFA concentration decreases over time from the initial value of 20 g/L sodium salt SCFA (equivalent to 14.6 g/L acetic acid, 15.4 g/L propionic acid and 16.0 g/L butyric acid) to below 5 g/L, specifically 4.3 g/L acetic acid, 4.4 g/L propionic acid and 3.6 g/L butyric acid. The volume of 5 M SCFA fed increases continuously with fermentation time to control the pH at 6.0, reaching 98.5 mL for acetic acid, 55.0 mL for propionic acid and 56.8 mL for butyric acid by 46.5 h, evidencing a dramatically different utilization rate for acetic acid, approaching utilization values almost double that of propionic and butyric acid. The fermentations were stopped at this point prior to full depletion of the 5 M acetic acid feed. Final cell dry weight concentrations of 25.7 ± 0.0 g/L, 31.3 ± 1.0 g/L and 29.5 ± 1.8 g/L were obtained for acetic acid, propionic acid and butyric acid pH-stat bioprocess. Considering the large difference in substrate utilization but comparable cell dry weight yields, a careful examination of titers, rates, and yields is merited.

### 3.4. Bioprocess Titer, Rate, Yield and Biomass Composition

The bioprocess parameters and resulting titer (*X*), rate (*R_X_*) and yield (*Y_X/S_*) for the pH-stat bioreactor processes are reported in Table 1 for the pH-stat bioprocess with sodium salt SCFA starting medium concentration and 5 M SCFA feed. Results for the pH-stat bioprocess with 10 g/L sodium salt SCFA starting medium concentration and 3 M SCFA feed are instead reported in Table A1. Rate is calculated as in Equation (1), where *t* is the fermentation time:

*R_X_* = *X*/*t*, (1)

Biomass yield is calculated as in Equation (2), from the batch volume (*V_Batch_*) and amount of SCFA consumed (*SCFA_consumed_*) on an acid-form basis by *C. jadinii* in the bioprocess calculated as in Equation (3) from the reported initial (*SCFA_Batch_*) and final (*SCFA_Final_*) concentrations, feed volumes (*V_Feed_*) and feed concentrations (*SCFA_Feed_*):

*Y_X/S_* = *X* × (*V_Batch_* + *V_Feed_*)/*SCFA_consumed_*, (2)

*SCFA_consumed_* = *SCFA_Batch_* × *V_Batch_* + *SCFA_Feed_* × *V_Feed_* − *SCFA_Final_* × (*V_Batch_* + *V_Feed_*). (3)

Due to achieving the highest biomass titer, while the other parameters in Equations (1)–(3) are the same or similar, the pH-stat bioprocess with propionate as a substrate also has the highest rate of 0.67 ± 0.02 g/L/h and yield of 0.36 ± 0.01 g/g, followed by butyrate and finally acetate. The yield of *C. jadinii* cultivated on propionate was found to be approximately 27% higher than the yield on butyrate, or approximately 45% higher than the yield on acetate. If instead the yield is calculated on a molar basis (gram biomass per mol SCFA) the difference is less pronounced. Molar yields are 14.8 ± 0.0 g/mol, 26.5 ± 0.9 g/mol and 24.8 ± 1.5 g/mol for acetate, propionate and butyrate, respectively. Propionate still stands out as one of the most efficient fermentation substrates even on a molar yield basis, being 79% higher than acetate, while not statistically significantly different from butyrate.

### 3.5. Single-Cell Protein Characterization

The SCP biomass was harvested at the end of fermentation by centrifugation, washed and freeze-dried, then accessed for crude protein, lipid and fatty acid content with the results reported in Table 2 for the pH-stat bioprocess with 20 g/L sodium SCFA and 5 M SCFA feed. The pH-stat bioprocess with propionate yielded the highest crude protein of 41.3% ± 0.0%, followed by butyrate at 38.9% ± 2.4% and the lowest being for acetate with 36.6% ± 2.8%. The crude lipid content shows the opposite trend, with acetate having the highest at 17.3%, followed by propionate at 15.1% and butyrate at 13.7%. The fatty acid composition of the lipids is also reported in Table 2, while similar results for the pH-stat bioprocess with 10 g/L sodium SCFA and 3 M SCFA feed are reported in Table A2.

The predominant fatty acids in the lipid fraction are palmitic acid (C16:0), oleic acid (C18:1), linoleic acid (C18:2) and linolenic acid (C18:3). The fatty acid profile exhibits a high degree of unsaturation, ranging from 81% of total lipids for propionate, up to 86% for butyrate, but with differences in the specific proportions of fatty acids. Especially interesting is the presence of odd-chain fatty in *C. jadinii* cultured on propionate, with 37% of the total fatty acids being odd-chain, mainly comprised of margaric acid (C17:0) and margaroleic acid (17:1), while especially oleic acid (C18:1) and linoleic acid (C18:2) contents are relatively diminished compared to *C. jadinii* cultured on acetate and butyrate.

## 4. Discussion

### 4.1. SCFA Metabolism in Cyberlindnera jadinii

To interpret our bioprocess performance, we first mapped SCFA uptake and catabolism in *C. jadinii*. Uptake of SCFA can occur both by passive diffusion and through mediated transport systems. Passive diffusion is expected to dominate at pH near or lower than the pKa of the specific SCFA, being 4.76 for acetic acid, 4.87 for propionic acid and 4.83 for butyric acid [41]. The monocarboxylate proton symport is shared by both acetate and propionate, as well as lactate and pyruvate, and is active over a wide pH range of 3.0 to 6.0 [42]. The genes encoding these transporters and their structures have recently been elucidated and characterized to recognize a wide range of mono-, di- and tricarboxylate substrates [43].

Once in the cell, SCFAs dissociate in the near neutral cytosol, potentially lowering cytosolic pH below the normal physiological range and triggering an energetically costly stress response [41]. Otherwise, acetate is converted to acetyl-CoA, entering either the tricarboxylic acid (TCA) cycle in the mitochondrion for energy generation, or glyoxylate cycle (GYC) in the peroxisome [44]. Alternatively, cytosolic acetyl-CoA can be converted to malonyl-CoA, and used for even-chain fatty acid synthesis (EC-FAS) and elongation in the endoplasmic reticulum, leading to even-chain fatty acids [45]. A free fatty acid (FFA) is added to lysophosphatidic acid (LPA) to form phosphatidic acid (PA). PA contains two fatty acids, one saturated and one unsaturated, and is the a precursor to the major membrane phospholipids in yeasts: phosphatidylinositol (PI), phosphatidylcholine (PC), phosphatidylserine (PS), and phosphatidylethanolamine (PE) [46]. Alternatively, the phosphate group can be removed from PA, converting it to diacylglycerol (DAG), which can have a third FFA added to form triacylglycerol (TAG). TAGs are stored in lipid droplets within the cell, and can be utilized as an energy reserve via β-oxidation under nutrient limitation [47].

In the related yeast, *Candida tropicalis*, butyrate is first converted to butyryl-CoA, then transformed through several steps into acetyl-CoA via β-oxidation in the peroxisome, also generating FADH_2_ and NADH [23,48]. We hypothesize that butyrate is metabolized in this way in *C. jadinii*, given that it can grow on butyrate as the sole carbon source [49]. Acetyl-CoA derived from butyrate then enters the TCA or GYC cycle, or EC-FAS, in a similar manner as described for acetate.

**Figure 4:**
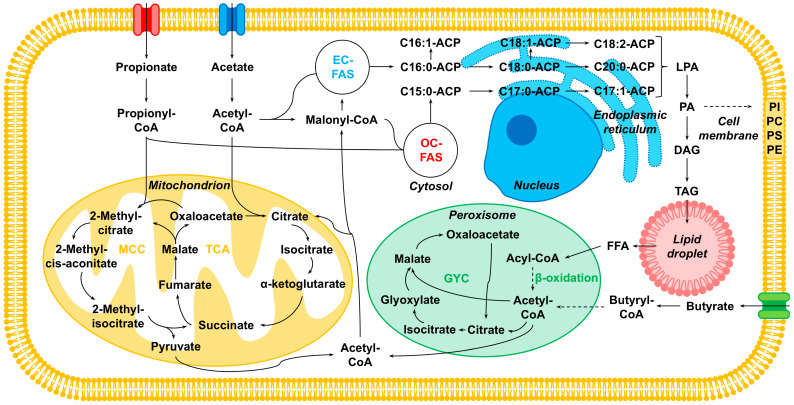
Metabolic pathways of acetate, propionate and butyrate utilization in *Cyberlindnera jadinii*. The solid and dashed arrows indicate single and multiple steps, respectively. Abbreviations: methylcitrate cycle (MCC), tricarboxylic acid cycle (TCA), glyoxylate cycle (GYC), even-chain fatty acid synthesis (EC-FAS), odd-chain fatty acid synthesis (OC-FAS), co-enzyme A (CoA), acyl carrier protein (ACP), lysophosphatidic acid (LPA), phosphatidic acid (PA), diacylglycerol (DAG), triacylglycerol (TAG), free fatty acid (FFA), phosphatidylinositol (PI), phosphatidylcholine (PC), phosphatidylserine (PS), and phosphatidylethanolamine (PE).

Propionate is first converted into propionyl-CoA, but as an odd-chain SCFA does not undergo β-oxidation, nor can it directly enter the TCA or GYC cycles. Instead, propionyl-CoA is metabolized through the closely related methylcitrate cycle (MCC), producing succinate and pyruvate, which are both capable of entering the TCA cycle [8]. The MCC is conserved in all kingdoms and is primarily considered a detoxification pathway, as propionyl-CoA is a potent cell growth inhibitor interfering with enzymes critical in the mitochondrial energy metabolism [50,51]. Alternatively, propionyl-CoA can be used as the building block for odd-chain fatty acid synthesis (OC-FAS) through repeated two-carbon chain elongation with malonyl-CoA [18,52]. Figure 4 illustrates the metabolic pathways for acetate, propionate and butyrate utilization in *C. jadinii*.

### 4.2. Single-Cell Protein from Short-Chain Fatty Acids

The growth rate and inhibition of *C. jadinii* cultivated on SCFAs has previously been investigated by Elmaleh et al., finding maximum SGR in absence of substrate inhibition of 0.5 h^−1^ on acetate, 0.29 h^−1^ on propionate and 0.5 h^−1^ on butyrate at pH 3.5 [49]. The maximum tolerable concentration of acetic acid was extrapolated to be 16 ± 2 g/L, and the growth rate linearly decreasing with substrate concentration in batch fermentation, the lowest measured being approximately 0.2 h^−1^ with 9.5 g/L acetic acid. The maximum tolerable concentration of propionic acid and butyric acid and growth rates at specific concentrations of SCFA were not reported by the authors [49].

The maximum SGR reported here are lower, ranging from 0.11 ± 0.01 h^−1^ to 0.15 ± 0.03 h^−1^ for acetate and butyrate, and from 0.13 ± 0.01 h^−1^ to 0.21 ± 0.02 h^−1^ for propionate in concentrations ranging from 2.5 g/L to 10 g/L. Moreover, only propionate exhibits an obvious trend of lowered SGR at higher concentrations. However, these differences can be attributed to substrate inhibition at practical working concentrations, medium compositions, bioprocess conditions (such as pH), and other factors. Considering the sensitivity to substrate inhibition and possible ion accumulation, this underlines the potential of pH-stat methods utilizing undissociated acid for both substrate and pH control.

The biomass yield of different carbon sources can be evaluated through the Gibbs energy dissipation per amount of produced biomass, which is simply correlated with the carbon chain length and degree of reduction in the carbon source [53]. This approach predicts that yields decrease for carbon sources with shorter chain lengths than 6 carbon atoms, and for degrees of reduction both higher and lower than 4, but does not explicitly take into account the different metabolic pathways for different substrates and microorganisms [53]. These considerations predict the highest yield for butyrate, followed by propionate and acetate, however the calculated molar yields (gram biomass per mol SCFA) are the highest for propionate at 26.5 ± 0.9 g/mol, compared to 24.8 ± 1.5 g/mol for butyrate and 14.8 ± 0.0 g/mol for acetate, indicating that propionate is particularly efficiently metabolized in *C. jadinii*.

The cultivation of *C. jadinii* on acetate and butyrate has previously been investigated and compared to other carbon sources and yeasts such as *Saccharomyces cerevisiae* by Verduyn et al. [54,55]. A yield of 0.39 g/g from acetate and 0.61 on butyrate was found for *C. jadinii* in carbon-limited chemostat cultures at dilution rate of 0.10 h^−1^ [54]. This is much higher than for the conventional yeast *S. cerevisiae*, with a yield of only 0.29 g/g from acetate, attributed to a higher efficiency of respiration, referred to as the phosphate/oxygen (P/O) ratio of 2.0 for *C. jadinii*, compared to 1.5 for *S. cerevisiae* [55]. Notably the growth on acetate is energy-limited, and enhanced yields can also be obtained through the addition of a co-substrate, such as formate [55].

The mitochondrial respiratory chain of yeasts varies significantly between genera, influencing their P/O ratio [55]. *S. cerevisiae* lacks the genetic information to produce the multi-subunit proton-pumping NADH dehydrogenase (i.e., Complex I) and relies solely on single-subunit, non-proton-translocating NADH dehydrogenases [56]. In contrast, *C. jadinii* possesses genes for both Complex I and non-proton-translocating NADH dehydrogenases. *C. jadinii* can therefore achieve higher efficiency of the electron transport chain by distributing NADH oxidation between Complex I and other systems [55].

The yield and protein-content of SCP obtained from fed-batch processes are typically lower than for chemostat processes, for example *C. utilis* cultured on ethanol in chemostat culture obtained a maximum yield of 0.83 g/g and protein content of 54%, however a similar fed-batch process had a biomass yield only of 0.65 g/g and protein content of 42% [57]. The results reported here for acetate and butyrate follow these trends, with the titer, rate and yield increasing from acetate to butyrate. The crude protein content also shows an increasing trend from acetate to butyrate, with a concurrent decrease in crude lipids. However, propionate does not follow the expected trend, having the highest titer, rate and yield, concurrent with a high protein content and unique lipid composition.

### 4.3. Lipid Production from Short-Chain Fatty Acids

*C. jadinii* is not considered as one of the approximately 70 species of wild-type yeast denoted as oleaginous, such as *Yarrowia lipolytica* and *Rhodosporidium toruloides*, which are capable of accumulating in excess of 20% intracellular lipids [58,59,60]. However, it has been investigated in the context of lipid production, on account of utilizing a wide range of carbon sources (including glucose, xylose, cellobiose, mannose, sucrose and glycerol), and having strong tolerance to fermentation inhibitors, such as furfurals and acetic acid [27,28]. The lipid contents and fatty acid profiles of *C. jadinii* cultivated on a variety of substrates have been previously reported: glucose [61,62,63], ethanol [57], glycerol [24], lignocellulosic sugars [29,64], wort broth [65,66], vinasse [61,67], tubers waste [68] and cellulose-derived cocktail of lactate, acetate and ethanol [32].

The lipid contents of *C. jadinii* range from as low as 0.3 wt% [69] to 7.9 wt% [32], with typical values around 6 wt% to 7 wt% [65,66,70]. Phospholipids such as phosphatidylcholine (PC) and phosphatidylethanolamine (PE) are the dominant lipid fraction with 65% to 75% of total fatty acids, and the remainder consisting of neutral lipids including triacylglycerols (TG), diacylglycerols (DG) and free fatty acids (FFA) [66]. The lipids contain primarily palmitic acid (C16:0), stearic acid (C18:0), oleic acid (C18:1), linoleic acid (C18:2) and linolenic acid (C18:3), while the total lipid content and the fatty acid profile depend on cultivation conditions, such as carbon-to-nitrogen ratio [71] and fermentation temperature [65,66].

*C. jadinii* can be cultivated on SCFAs as the sole carbon source [32,49], although weak organic acids do have an uncoupling effect on cellular respiration [72,73]. Previously a lipid content of 7.9 wt% was reported for *C. jadinii* cultivated on a cellulose-derived cocktail of lactate, acetate and ethanol [32]. The fatty acid profile of the lipids consisted of 14% palmitic acid (C16:0), 2% stearic acid (C18:0), 21% oleic acid (C18:1), 42% linoleic acid (C18:2), 17% linolenic acid (C18:3) and 4% other fatty acids, with unsaturated fatty acids making up 83% of the total [32].

Compared to the literature, much higher lipid contents were obtained in *C. jadinii* cultivation on acetate (17.3%), propionate (15.1%) and butyrate (13.7%), approaching the 20 wt% lipid content that defines oleaginous yeasts [58]. Possibly this indicates TAG accumulation in lipid droplets in *C. jadinii* cultivated on these substrates. The lipid analysis through FAME derivatization conducted in this study is unable to differentiate between TAG and phospholipids, but future studies could employ mass spectrometry-based lipidomics methods to differentiate these lipid classes [74].

The fatty acid profile when cultivated on acetate and butyrate were broadly similar, containing the same five prominent fatty acids, with minor amounts of C16:1. The major difference is a higher proportion of C18:1 ranging from 31% to 41%, more variable C18:2 ranging from 29% to 51%, and lower proportion of C18:3 ranging from 4% to 11%. The overall proportion of unsaturated fatty acids however was similarly high for *C. jadinii* cultivated on acetate (84%), propionate (81%) and butyrate (86%), which is valuable for use in animal feed, as the unsaturated fatty acids transfer into meat, milk and eggs improving their nutritional value [33].

The lipid fraction of *C. jadinii* cultivated on propionate contains 63% even-chain fatty acids in similar relative proportions, but also 37% odd-chain fatty acids, mainly margaric acid (C17:0) and margaroleic acid (C17:1) with a minor component of pentadecanoic acid (C15:0). Odd-chain fatty acids have previously been found in *C. jadinii* cultivated on three-carbon atom glycerol, but in low amounts of 7% to 9% of only margaric acid (C17:0) [70]. We believe this is the first report of significant amounts of odd-chain fatty acids in *C. jadinii*, warranting a more in-depth look at the production potential.

### 4.4. C. jadinii as a Chassis for Odd-Chain Fatty Acid Production

Propionic acid is an effective anti-microbial at concentrations of 0.1–1%, and is used as a preservative in food, beverage and bakery products [75,76]. Evidently *C. jadinii* has significant resistance to the toxicity of propionate and propionyl-CoA in view of the high biomass yields and limited inhibition observed at concentrations over 15 g/L seen here.

In contrast, the conventional yeast *Saccharomyces cerevisiae* cannot grow on propionate as the sole carbon source, only with co-substrates such as glucose or methylmalonate [77,78,79]. Even sub-inhibitory concentrations (4.7 g/L) of propionate in *S. cerevisiae* leads to endocytosis, alterations in the cell cycle, and impaired of cellular respiration [80,81]. The causes of propionate toxicity in *S. cerevisiae* have been hypothesized to be: intracellular acidification [82]; toxicity of methylcitrate in the peroxisomes [78]; and possible lack of pyruvate transport out of the mitochondrion [83]. Intracellular acidification and maintenance of pH homeostasis applies to SCFAs in general [22], and acetic acid and butyric acid are also reported to exhibit a lower degree of toxicity to *S. cerevisiae* [84,85,86].

Odd-chain fatty acid production from propionate has been evidenced in a number of yeast species, including *Candida* spp., *Trichosporon cutaneum*, *Rhodococcus opacus* and *Yarrowia lipolytica* [18]. Propionic acid is a common carbon source to induce odd-chain fatty acid accumulation in yeasts, with up to 15 g/L being used, typically with a co-substrate to improve utilization for fatty acid synthesis [18]. As much as 85.1% odd-chain fatty acids have been reported with wild-type yeasts, comprising C15:0, C17:0 and C17:1, in titers up to 1.19 g/L in *Rhodococcus* sp. [87].

These yeasts are still susceptible to propionate toxicity, for example the growth of *Yarrowia lipolytica* is inhibited in concentrations higher than 5 g/L [18]. Fed-batch, co-substrate and genetic engineering strategies have been employed to mitigate toxicity issues [19,88,89]. Such approaches have led to an odd-chain fatty acid proportion of 62.1% and titer of 1.87 g/L in genetically engineered *Y. lipolytica* [90]. Notably CRISPR-Cas9-based genome editing tools have recently been developed for *C. jadinii* [91], opening the potential for improving odd-chain fatty acid production via genetic engineering.

Recent work by Bonzanini et al. screened 19 wild-type yeast species for the ability to grow on propionic acid as the sole carbon source in concentrations between 5 g/L and 29 g/L [92]. Only eight yeast strains exhibited growth, yielding 37% to 89% odd-chain fatty acids, with *Cutaneotrichosporon oleaginosus* highlighted as the best odd-chain fatty acid producer with a titer of 0.94 g/L and yield of 0.07 g/g [92].

In comparison to these, *C. jadinii* is able to tolerate over 15 g/L propionate, and capable of utilizing it as a sole carbon and energy source while producing odd-chain fatty acids. Bioprocess titer, rate and yield (TRY) for odd-chain fatty acid production can readily be calculated from Table 1 and Table 2 to be 1.74 ± 0.06 g/L, 0.037 ± 0.001 g/L/h and 0.020 ± 0.001 g/g, respectively. The TRY for *C. jadinii* compare favorably to other odd-chain fatty acid producing yeasts in the literature, and in fact to the best of our knowledge the titer obtained in the pH-stat bioprocess is the highest reported for any wild-type yeast.

## 5. Conclusions

The non-conventional yeast *Cyberlindnera jadinii* is not only tolerant to short-chain fatty acids, but capable of efficiently utilizing them as the carbon and energy source for single-cell protein and lipid production. Fed-batch pH-stat bioprocesses were developed for acetate, propionate and butyrate utilization. *C. jadinii* cultured on propionate had the highest biomass titer of 31.3 ± 1.0 g/L, rate of 0.67 ± 0.02 g/L/h and yield of 0.36 ± 0.01 g/g, while the biomass comprised a crude protein content of 41.3% ± 0.0% and crude lipid content of 15.1%. Slightly lower values were obtained for cultivations on butyrate, with titer of 29.5 ± 1.8 g/L, rate of 0.64 ± 0.04 g/L, yield of 0.28 ± 0.02 g/g, crude protein of 38.9% ± 2.4% and crude lipid content of 13.7%. This work showcases the potential of nutritious *C. jadinii* to be produced from non-sugar substrates for use as a single-cell protein ingredient in alternative food and feed applications.

The lipids of *C. jadinii* cultured on acetate, propionate and butyrate contained 81% to 86% unsaturated fatty acids, dominated by oleic acid (C18:1) and linoleic acid (C18:2). *C. jadinii* fed acetate and butyrate only biosynthesized even-chain fatty acids, while propionate led to the accumulation of 37% odd-chain fatty acids, primarily margaric acid (C17:0) and margaroleic acid (C17:1). The titer, rate and yield of odd-chain fatty acid production in the fed-batch pH-stat bioprocess of 1.74 ± 0.06 g/L, 0.037 ± 0.001 g/L/h and 0.020 ± 0.001 g/g appear to be the highest achieved in the literature for a wild-type yeast, showcasing the potential of *C. jadinii* as a chassis for odd-chain fatty acid production.

Further work is suggested to investigate the proportion of triacylglycerols and phospholipids in *C. jadinii* cultured on short-chain fatty acids using methods such as mass spectroscopy-based lipidomics. Strategies such as co-feeding and genetic engineering may be used to improve the odd-chain fatty acid production in *C. jadinii* to reach commercially relevant productivity. An especially compelling direction for future research is the use of the acidogenic phase of anaerobic digestion of organic waste as an economical and sustainable source of short-chain fatty acids for single-cell protein and odd-chain fatty acid production.

## Figures and Tables

**Table 1:** Titer, rate and yield (TRY) for utilization of short-chain fatty acids (SCFAs) in single-cell protein (SCP) production of *Cyberlindnera jadinii* on dry weight basis (dwb) in pH-stat fermentations.

Fed-Batch pH-Stat Fermentation 20 g/L Sodium SCFA, 5 M SCFA Feed			
pH-stat parameters (dwb)	Acetate	Propionate	Butyrate
pH set-point	6.0 ± 0.1	6.0 ± 0.1	6.0 ± 0.1
Time (*t*)	46.5 h	46.5 h	46.5 h
Batch volume (*V_Batch_*)	200 mL	200 mL	200 mL
Feed volume (*V_Feed_*)	98.5 mL	55.0 mL	55.5 mL
Batch concentration (*SCFA_Batch_*)	14.6 g/L	15.4 g/L	16.0 g/L
Feed concentration (*SCFA_Feed_*)	300 g/L	370 g/L	441 g/L
Final concentration (*SCFA_Final_*)	4.32 g/L	4.42 g/L	3.56 g/L
pH-stat TRY (dwb)	Acetate	Propionate	Butyrate
Biomass titer (*X*)	25.7 ± 0.0 g/L	31.3 ± 1.0 g/L	29.5 ± 1.8 g/L
Biomass rate (*R_X_*)	0.54 ± 0.00 g/L/h	0.67 ± 0.02 g/L/h	0.64 g/L/h ± 0.04
Biomass yield (*Y_X/S_*)	0.25 ± 0.00 g/g	0.36 ± 0.01 g/g	0.28 ± 0.02 g/g

**Table 2:** Biomass composition for utilization of short-chain fatty acids (SCFAs) in single-cell protein (SCP) production of *Cyberlindnera jadinii* on dry weight basis (dwb) in pH-stat fermentations.

Fed-Batch pH-Stat Fermentation 20 g/L Sodium SCFA, 5 M SCFA Feed			
*C. jadinii* composition (dwb)	Acetate	Propionate	Butyrate
Crude protein	36.6% ± 2.8%	41.3% ± 0.0%	38.9% ± 2.4%
Crude lipid	17.3%	15.1%	13.7%
*C. jadinii* fatty acids (dwb)	Acetate	Propionate	Butyrate
Pentadecanoic acid (C15:0)	0%	1%	0%
Palmitic acid (C16:0)	15%	9%	10%
Palmitoleic acid (C16:1)	2%	2%	1%
Margaric acid (C17:0)	0%	9%	0%
Margaroleic acid (C17:1)	0%	27%	0%
Stearic acid (C18:0)	2%	1%	3%
Oleic acid (C18:1)	41%	26%	31%
Linoleic acid (C18:2)	29%	20%	51%
Linolenic acid (C18:3)	11%	6%	4%
Total saturated	16%	19%	14%
Total unsaturated	84%	81%	86%
Total odd-chain	0%	37%	0%
Total even-chain	100%	63%	100%

## Data Availability

The original contributions presented in this study are included in the article. Further inquiries can be directed to the corresponding authors.

## Appendix A

### Fed-Batch pH-Stat Fermentation 10g/L Sodium SCFA and 3M SCFA Feed

**Table A1:** pH-stat parameters, titer, rate and yield (TRY) for utilization of short-chain fatty acids (SCFAs) in single-cell protein (SCP) production of *Cyberlindnera jadinii* on dry weight basis (dwb).

Fed-Batch pH-Stat Fermentation 10 g/L Sodium SCFA, 3 M SCFA Feed			
pH-stat parameters (dwb)	Acetate	Propionate	Butyrate
pH set-point	6.0 ± 0.1	6.0 ± 0.1	6.0 ± 0.1
Time (*t*)	48 h	48 h	48 h
Batch volume (*V_Batch_*)	200 mL	200 mL	200 mL
Feed volume (*V_Feed_*)	100 mL	100 mL	100 mL
Batch concentration (*SCFA_Batch_*)	7.3 g/L	7.7 g/L	8.0 g/L
Feed concentration (*SCFA_Feed_*)	180 g/L	222 g/L	264 g/L
Final concentration (*SCFA_Final_*)	0 g/L	0 g/L	0 g/L
pH-stat TRY (dwb)	Acetate	Propionate	Butyrate
Biomass titer (*X*)	14.3 ± 0.12 g/L	17.1 ± 0.1 g/L	15.7 ± 0.2 g/L
Biomass rate (*R_X_*)	0.30 ± 0.00 g/L/h	0.36 ± 0.00 g/L/h	0.33 ± 0.00g/L/h
Biomass yield (*Y_X/S_*)	0.22 ± 0.00 g/g	0.22 ± 0.00 g/g	0.17 ± 0.00 g/g

**Table A2:** Biomass composition for utilization of short-chain fatty acids (SCFAs) in single-cell protein (SCP) production of *Cyberlindnera jadinii* on dry weight basis (dwb). Crude protein content of SCP from propionate and butyrate was not measured.

Fed-Batch pH-Stat Fermentation 10 g/L Sodium SCFA, 3 M SCFA Feed			
*C. jadinii* composition (dwb)	Acetate	Propionate	Butyrate
Crude protein	41.8% ± 0.7%	-	-
Crude lipid	11.50%	13.50%	12.50%
*C. jadinii* fatty acids (dwb)	Acetate	Propionate	Butyrate
Pentadecanoic acid (C15:0)	0%	1%	0%
Palmitic acid (C16:0)	11%	6%	9%
Palmitoleic acid (C16:1)	1%	1%	1%
Margaric acid (C17:0)	0%	7%	0%
Margaroleic acid (C17:1)	0%	32%	0%
Stearic acid (C18:0)	1%	0%	2%
Oleic acid (C18:1)	33%	22%	35%
Linoleic acid (C18:2)	43%	24%	46%
Linolenic acid (C18:3)	10%	7%	7%
Total saturated	12%	13%	11%
Total unsaturated	88%	87%	89%
Total odd-chain	0%	39%	0%
Total even-chain	100%	61%	100%
